# Biomechanical adaptations of kicks following vertical and single-leg plyometric training in competitive Wushu sanda athletes: implications for lower-limb explosive power and balance

**DOI:** 10.3389/fphys.2026.1888868

**Published:** 2026-07-03

**Authors:** Guo Cheng, Gang Chen, Na Zang, Changwei Chen, Jianxin Zhou, Peng Liu

**Affiliations:** 1 School of Physical Education, Nanjing Xiaozhuang University, Nanjing, China; 2 Nanjing University of Finance & Hongshan College, Nanjing, China; 3 Basic Courses Department, Qilu Institute of Technology, Jinan, China

**Keywords:** biomechanics, dynamic balance, explosive power, kicking performance, plyometric training, Wushu sanda

## Abstract

**Background:**

Kicking techniques are fundamental components of performance in competitive Wushu Sanda, requiring a combination of lower-limb explosive power and dynamic balance to generate high-velocity strikes while maintaining postural stability. Plyometric training is widely recognized for enhancing neuromuscular performance; however, limited research has examined how different plyometric modalities influence the biomechanics of sport-specific kicking movements in martial arts athletes.

**Purpose:**

This study aimed to investigate the biomechanical adaptations of kicking performance following vertical plyometric training (VPT) and single-leg plyometric training (SLPT) in competitive Wushu Sanda athletes.

**Methods:**

Thirty-six competitive Wushu Sanda athletes (18–25 years) were randomly assigned to three groups: VPT (n = 12), SLPT (n = 12), and a control group (CG; n = 12). The intervention lasted eight weeks with training sessions conducted three times per week. Biomechanical assessments were performed before and after the intervention using a three-dimensional motion capture system (250 Hz) and a force platform (1000 Hz). Key variables included peak ground reaction force (GRF), rate of force development (RFD), kick velocity, hip and knee angular velocities, and center of pressure (COP) displacement. Data were analyzed using two-way repeated-measures ANOVA (Group × Time), with significance set at p < 0.05.

**Results:**

Significant group × time interactions were observed for peak GRF (p < 0.001), RFD (p = 0.001), kick velocity (p = 0.001), knee angular velocity (p = 0.003), and COP displacement (p < 0.001). The VPT group demonstrated greater improvements in explosive power and kick velocity, whereas the SLPT group exhibited superior improvements in dynamic balance and inter-limb stability.

**Conclusion:**

Both plyometric training modalities significantly enhanced kicking biomechanics in Wushu Sanda athletes, but produced distinct neuromuscular adaptations. VPT was more effective for improving explosive power and kicking velocity, while SLPT better enhanced dynamic balance. Integrating both modalities may optimize sport-specific performance in martial arts athletes.

## Introduction

Wushu is an umbrella term for Chinese martial arts that includes multiple competitive disciplines, most notably Taolu and Sanda. Taolu emphasizes choreographed routines, technical execution, and aesthetic movement quality, whereas Sanda is a full-contact combat discipline characterized by striking, kicking, throwing, and defensive actions. The present study specifically focuses on Wushu Sanda because kicking techniques are central to both scoring performance and tactical effectiveness in this discipline. Successful kicking execution in Sanda requires athletes to generate high levels of lower-limb explosive power while simultaneously maintaining dynamic balance and postural stability (Jlid et al., 2016; Xu et al., 2025). From a biomechanical perspective, kicking performance depends on the coordinated transfer of mechanical energy along the lower-limb kinetic chain, involving the hip, knee, and ankle joints, which ultimately determines the velocity, force, and accuracy of the kick (Corcoran et al., 2024; Jung and Park, 2022).

Lower limb explosive power has been widely recognized as a key determinant of performance in striking-based martial arts such as taekwondo, karate, and Wushu (Hidayat et al., 2026; Maffiuletti et al., 2016; Sánchez-ramírez et al., 2025). Athletes with higher explosive capabilities are able to generate faster limb acceleration and greater impact force during kicking actions (Corcoran et al., 2024; Maffiuletti et al., 2016). Biomechanically, explosive movements are closely associated with the efficiency of the stretch shortening cycle (SSC), which enables muscles and tendons to store elastic energy during the eccentric phase and rapidly release it during the concentric phase (Komi, 2000). This neuromuscular mechanism enhances the rate of force development (RFD) and contributes to improved movement velocity and power production (Aagaard et al., 2026; Cormie et al., 2013).

Plyometric training has been extensively used as an effective method to improve explosive power in athletes due to its ability to optimize the stretch shortening cycle and neuromuscular coordination (Seiberl et al., 2015). Through repeated rapid eccentric concentric muscle actions, plyometric exercises enhance muscle tendon stiffness, motor unit recruitment, and intermuscular coordination, leading to improved force generation and movement efficiency (Markovic and Mikulic, 2010; Slimani et al., 2016). Numerous studies across different sports disciplines have demonstrated that plyometric training can significantly improve vertical jump performance, sprint speed, and lower limb power (E. S. De Villarreal et al., 2010). Consequently, plyometric exercises are frequently incorporated into strength and conditioning programs for sports in which explosive movements play a core functional role, particularly combat sports that require rapid striking, kicking, and dynamic postural control (Davies et al., 2015; E. S.-S. De Villarreal et al., 2009).

Among the various forms of plyometric exercises, VPT such as squat jumps, countermovement jumps, and box jumps has been widely applied to develop bilateral explosive power (Moran et al., 2023). These exercises primarily target vertical force production and enhance the RFD in the lower extremities. Previous research has shown that VPT can significantly increase peak GRF, jump performance, and sprint acceleration (Wallace et al., 2010). However, despite these well-documented benefits, most studies have primarily focused on general athletic performance indicators rather than examining how such training adaptations influence sport-specific technical movements such as kicking in martial arts (Ramirez-Campillo et al., 2022).

In contrast, SLPT has recently gained attention due to its potential to improve unilateral strength, neuromuscular control, and dynamic balance (Drouzas et al., 2020; Ramirez-Campillo et al., 2022). Many sport-specific actions including kicking techniques in Wushu are inherently unilateral movements, where athletes must maintain stability on a supporting leg while producing high-speed movements with the kicking leg (Gavagan and Sayers, 2017). SLPT impose greater demands on proprioceptive control, joint stabilization, and interlimb coordination, which may lead to improved balance and movement efficiency during complex motor tasks (Cao et al., 2024). Studies conducted in sports such as soccer, basketball, and taekwondo have demonstrated that unilateral plyometric training can improve dynamic balance, reduce limb asymmetries, and enhance functional performance. Nevertheless, the specific biomechanical adaptations associated with SLPT in martial arts kicking techniques remain insufficiently explored (Drouzas et al., 2020; Ramirez-campillo et al., 2021).

Despite the growing body of literature on plyometric training and sports performance, several limitations remain evident in current research (Gül et al., 2019). First, the majority of existing studies have focused primarily on general performance outcomes such as jump height, sprint speed, and muscular strength, while relatively few investigations have examined detailed biomechanical changes in sport-specific movements. Second, limited research has directly compared different plyometric training modalities particularly vertical versus unilateral plyometric training in relation to kicking performance in martial arts athletes (Carvalho et al., 2021). These two training approaches may produce different neuromuscular and biomechanical adaptations due to differences in force production patterns and balance requirements. Third, previous studies have often examined explosive power and balance as separate performance components. However, during sport-specific kicking movements, explosive force generation and postural control occur simultaneously and jointly influence movement effectiveness (Sant’ Ana et al., 2021).

Understanding the interaction between explosive power development and balance control during sport-specific movements is therefore essential for optimizing training strategies in martial arts athletes. From a biomechanical standpoint, kicking performance is influenced not only by the magnitude of force production but also by movement coordination, joint angular velocity, and postural stability during the kicking phase. Investigating how different plyometric training modalities influence these biomechanical factors may provide deeper insight into the mechanisms underlying improved kicking performance.

Therefore, this study aims to investigate the biomechanical adaptations of kicking performance following VPT and SLPT in competitive Wushu Sanda athletes. Specifically, this study examines how these two plyometric training modalities influence lower-limb explosive power, dynamic balance, and biomechanical variables associated with kicking execution, including joint kinematics, force production, and peak kicking velocity. By comparing the effects of VPT and SLPT, this research seeks to provide evidence-based insights for optimizing strength and conditioning programs designed to enhance sport-specific performance in competitive Wushu Sanda athletes. Based on the biomechanical demands of Wushu Sanda kicking and the distinct neuromuscular characteristics of bilateral and unilateral plyometric training, it was hypothesized that both VPT and SLPT would produce greater improvements in Sanda-specific kicking biomechanics than regular Wushu Sanda training alone. Furthermore, VPT was hypothesized to produce greater improvements in explosive force-production and speed-related variables, including peak GRF, RFD, peak kicking velocity, and peak hip and knee angular velocities, whereas SLPT was hypothesized to produce greater improvements in unilateral balance and neuromuscular control, as reflected by reduced COP displacement and improved inter-limb stability.

## Research methods

### Research design

This study employed a three-arm, parallel-group randomized controlled experimental design with repeated measures to investigate the biomechanical adaptations of kicking performance following two plyometric training modalities: VPT and SLPT. Participants were randomly assigned to three groups: VPT, SLPT, and a CG that continued regular Wushu Sanda training without additional plyometric intervention. The intervention lasted eight weeks, with training sessions conducted three times per week. Pre-test and post-test assessments were performed to evaluate changes in lower limb explosive power, dynamic balance, and biomechanical characteristics of kicking performance.

Biomechanical testing was conducted under standardized laboratory conditions. Participants performed standardized Wushu Sanda kicking tasks while kinematic and kinetic data were recorded using a high-speed motion capture system and force platform. These measurements allowed the analysis of joint movement patterns, peak kicking velocity, and peak GRF during kick execution. The testing protocol was designed to enable systematic comparison of biomechanical and performance outcomes across the VPT, SLPT, and CG groups at pre-test and post-test. This design allowed the examination of both within-group training adaptations and between-group differences under standardized experimental conditions.

### Participants

A total of 36 competitive Wushu Sanda athletes (male and female) voluntarily participated in this study. Participants were recruited from university Wushu Sanda teams and regional training centers. All athletes had at least three years of systematic training experience and were actively involved in regular training and competition. The inclusion criteria were as follows: (1) aged 18–25 years, (2) a minimum of three years of competitive Wushu Sanda training, (3) participation in regular training at least four times per week, and (4) no history of lower-limb musculoskeletal injury within the previous six months. Athletes were excluded if they had neurological, orthopedic, or cardiovascular conditions that could affect their performance or ability to complete the training intervention. An *a priori* power analysis was conducted using G*Power software (version 3.1) to determine the minimum sample size required for this study. The calculation was based on a repeated-measures ANOVA (within–between interaction) corresponding to the study design of three groups and two measurement points (pre-test and post-test). The parameters used were an effect size (f) of 0.25, a significance level (α) of 0.05, and a statistical power (1 − β) of 0.80, with an assumed correlation among repeated measures of 0.50.

The analysis indicated that a minimum of 30 participants was required to detect a statistically significant interaction effect. To account for potential participant dropout, the sample size was increased to 36 athletes. Participants were randomly assigned to one of three groups: VPT; n = 12, SLPT; n = 12, and CG; n = 12 that continued their regular Wushu Sanda training without additional plyometric intervention. Prior to participation, all athletes were informed about the study procedures, potential risks, and benefits. Ethical approval was obtained from the Research Ethics Committee of the School of Physical Education, Nanjing Xiaozhuang University (Approval No. XZ2026025). All procedures were conducted in accordance with the ethical principles outlined in the Declaration of Helsinki, and written informed consent was obtained from all participants.

### Training intervention

The training intervention was designed to investigate the effects of two distinct plyometric modalities on lower-limb explosive power, dynamic balance, and kicking biomechanics in competitive Wushu Sanda athletes. Participants in the experimental groups underwent either VPT or SLPT, while CG continued with their regular Wushu Sanda training. The plyometric training sessions were implemented by replacing the athletes’ usual conditioning component within regular Wushu Sanda training, rather than being added as extra training sessions. Therefore, total weekly training duration was kept comparable across the VPT, SLPT, and CG groups. The main difference among groups was the content of the conditioning component: bilateral vertical plyometric exercises in the VPT group, unilateral plyometric exercises in the SLPT group, and regular conditioning activities in the control group. The VPT program focused on bilateral exercises aimed at enhancing maximal vertical jump performance, reactive strength, and stretch-shortening cycle efficiency. In contrast, the SLPT program emphasized unilateral exercises to improve single-leg power, proprioception, and dynamic balance, reflecting the unilateral nature of kicking actions in Wushu Sanda. Training was performed three times per week for eight weeks, with progressive adjustments in intensity and complexity to ensure overload while minimizing fatigue and injury risk. **Table 1** provides a detailed overview of the exercise types, sets and repetitions, training focus, progression strategies, and rest intervals for each group. This structured approach allowed for a systematic comparison of the biomechanical adaptations induced by different plyometric training modalities in sport-specific movements.

**Table 1:** Training program.

Group	Exercise type	Exercises	Sets × repetitions	Focus/target	Progression	Rest between sets
VPT	Bilateral Plyometric	Squat Jumps	3–4 × 8–12	Maximal vertical jump, lower-limb explosive power, stretch-shortening cycle (SSC) efficiency	Increase jump height or add weighted vest every 2 weeks	60–90 s
		Countermovement Jumps (CMJ)	3–4 × 8–12	Rapid eccentric-concentric transitions, force development	Gradual increase in speed and jump amplitude	60–90 s
		Box Jumps	3–4 × 6–10	Reactive strength, peak GRF	Increase box height progressively	60–90 s
SLPT	Unilateral Plyometric	Single-Leg Hops	3–4 × 8–12 per leg	Dynamic balance, unilateral explosive power, proprioception	Increase distance or add instability (e.g., soft surface)	60–90 s
		Lateral Single-Leg Bounds	3–4 × 6–10 per leg	Mediolateral stability, interlimb coordination	Increase bounding distance gradually	60–90 s
		Single-Leg Box Jumps	3–4 × 6–10 per leg	Single-leg power, landing control	Increase box height progressively	60–90 s
CG	Regular Wushu Training	Standard technical and tactical training	As per routine	Maintain regular performance	Not applicable	Not applicable

### Experimental procedures

All participants underwent a standardized testing protocol before (pre-test) and after (post-test) the eight-week intervention. Testing was conducted under controlled laboratory conditions to ensure consistency and reliability of the biomechanical measurements (Jung and Park, 2018; Sørensen et al., 1996). Prior to data collection, athletes performed a 10-minute standardized warm-up consisting of dynamic stretching, joint mobility exercises, and low-intensity movement drills to minimize injury risk and optimize neuromuscular readiness (Davies et al., 2015). Participants then performed standardized Wushu Sanda kicking tasks representative of competitive performance, including front kicks, side kicks, and roundhouse kicks (Corcoran et al., 2024; Gavagan and Sayers, 2017; Sørensen et al., 1996). Each kick was executed at maximal effort while maintaining proper technique. Three successful repetitions were recorded for each kick type, with approximately 60 seconds of rest between trials to reduce fatigue effects (Gavagan and Sayers, 2017; Jung and Park, 2022).

To reduce inter-trial variability, verbal instructions and demonstrations were standardized, and a familiarization session was conducted before pre-test measurements (Jung and Park, 2022; Sørensen et al., 1996). The sequence of kicking tasks was randomized across participants to control for order effects. During each trial, kinematic and kinetic data were recorded simultaneously using a synchronized motion capture system and force platform. Participants were instructed to perform each movement as naturally as possible to replicate sport-specific conditions (Corcoran et al., 2024; Gavagan and Sayers, 2017). Data acquisition was completed within a single testing session at each time point. All experimental procedures were conducted in accordance with ethical standards, and participants were continuously monitored for safety.

### Biomechanical measurements

Biomechanical measurements were conducted to quantify the effects of the plyometric interventions on kicking performance in competitive Wushu Sanda athletes. Three-dimensional lower-limb kinematics were recorded using a high-speed motion capture system sampling at 250 Hz (Jung and Park, 2022; Sørensen et al., 1996). Reflective markers were placed on anatomical landmarks, including the anterior superior iliac spine, greater trochanter, lateral and medial femoral epicondyles, lateral and medial malleoli, and the fifth metatarsal head (Jung and Park, 2022; Sørensen et al., 1996). These markers enabled the calculation of hip, knee, and ankle joint angles and range of motion, as well as peak angular velocities of the hip and knee joints during kicking tasks. Kinetic data were captured simultaneously using a force platform sampling at 1000 Hz. The force platform was used to measure peak GRF and RFD during support phases (Maffiuletti et al., 2016; Wallace et al., 2010).

Peak kicking velocity was determined from the displacement of the distal foot marker over time, providing an indicator of lower-limb explosive performance during striking actions (Corcoran et al., 2024; Gavagan and Sayers, 2017; Sørensen et al., 1996). Dynamic balance was assessed during single-leg support phases using COP displacement and stability indices derived from force platform data (Bouteraa et al., 2020; Gavagan and Sayers, 2017). The primary biomechanical outcome variables were peak joint angles, peak hip and knee angular velocities, peak kicking velocity, peak GRF, RFD, and COP displacement. These variables were selected to provide a comprehensive assessment of explosive force production, lower-limb movement coordination, kicking speed, and dynamic balance following VPT and SLPT.

### Data processing and statistical analysis

For each participant, the mean value of three successful trials per kick type was calculated for each biomechanical variable. Kinematic and kinetic signals were filtered using a fourth-order Butterworth low-pass filter, with cut-off frequencies of 15 Hz for kinematic data and 50 Hz for kinetic data, to reduce noise while preserving movement-related signals (Jung and Park, 2022; Sørensen et al., 1996). Data were processed using customized motion analysis software, and normalized values were used where appropriate to account for inter-individual differences in body size and limb length.

Variables of interest were aggregated across kick types to provide an overall assessment of sport-specific kicking performance. Additionally, asymmetry indices were derived for unilateral variables to assess balance and inter-limb coordination. Data integrity and quality checks were performed before statistical analysis to identify and exclude trials with technical errors or outliers. All data processing and calculations were conducted by researchers blinded to group allocation to reduce potential bias.

All statistical analyses were performed using SPSS version 26.0 (IBM Corp., Armonk, NY, USA). Data are expressed as mean ± standard deviation (SD) for normally distributed variables. Normality of data distributions was assessed using the Shapiro–Wilk test, and homogeneity of variances was verified with Levene’s test. All outcome variables satisfied the assumptions for parametric analysis; therefore, no variables were analyzed using non-parametric tests. For variables that met parametric assumptions, a two-way repeated-measures ANOVA (Group × Time) was conducted to examine within-group changes (pre- vs. post-intervention) and between-group differences (VPT, SLPT, CG). Where sphericity assumptions were violated, the Greenhouse–Geisser correction was applied.

For non-normally distributed data, the non-parametric equivalent, the Friedman test, was used for within-group comparisons, and the Kruskal–Wallis test was used for between-group comparisons. Significant main effects were followed by *post-hoc* pairwise comparisons with Bonferroni correction to control for Type I error. Effect sizes were calculated to evaluate the magnitude of changes using Cohen’s d for within-group differences and partial eta squared (η^2^) for ANOVA results, interpreted according to conventional thresholds (small: 0.2, medium: 0.5, large: 0.8 for Cohen’s d; small: 0.01, medium: 0.06, large: 0.14 for η^2^). The level of statistical significance was set at p < 0.05. All analyses were conducted on a per-protocol basis, with participants included only if they completed the full training intervention and testing sessions. This statistical framework allowed for a robust evaluation of the differential effects of VPT and SLPT on biomechanical and performance variables in competitive Wushu Sanda athletes.

## Results

### Participant characteristics

A total of 36 competitive Wushu Sanda athletes completed the eight-week training intervention and were included in the final analysis (VPT = 12, SLPT = 12, CG = 12). No injuries or dropouts occurred during the intervention period. Baseline characteristics of the participants are presented in **Table 2**. No significant differences were observed among the three groups at pre-test for age, sex distribution, height, body mass, BMI, or training experience (p > 0.05), indicating successful randomization and comparable baseline conditions. These results confirm that participants across groups were homogeneous prior to the training intervention.

**Table 2:** Baseline characteristics of participants.

Variable	VPT (n = 12)	SLPT (n = 12)	CG (n = 12)	P-value
Age (years)	21.3 ± 1.9	21.6 ± 2.1	21.1 ± 2.0	0.82
Sex, male/female (n)	7/5	6/6	7/5	0.89
Height (cm)	172.4 ± 6.5	173.1 ± 7.2	171.9 ± 6.8	0.89
Body mass (kg)	66.2 ± 7.8	67.4 ± 8.1	65.8 ± 7.5	0.87
BMI (kg/m^2^)	22.3 ± 1.8	22.5 ± 1.9	22.2 ± 1.7	0.88
Training experience (years)	6.4 ± 1.7	6.2 ± 1.8	6.5 ± 1.6	0.91

Values are presented as mean ± SD unless otherwise indicated. VPT = Vertical Plyometric Training; SLPT = Single-Leg Plyometric Training; CG = Control Group; BMI = body mass index. Sex distribution was compared among groups using the chi-square test, while continuous variables were compared using one-way ANOVA. No significant baseline differences were observed among groups (p > 0.05).

### Changes in lower-limb explosive power

**Figure 1:**
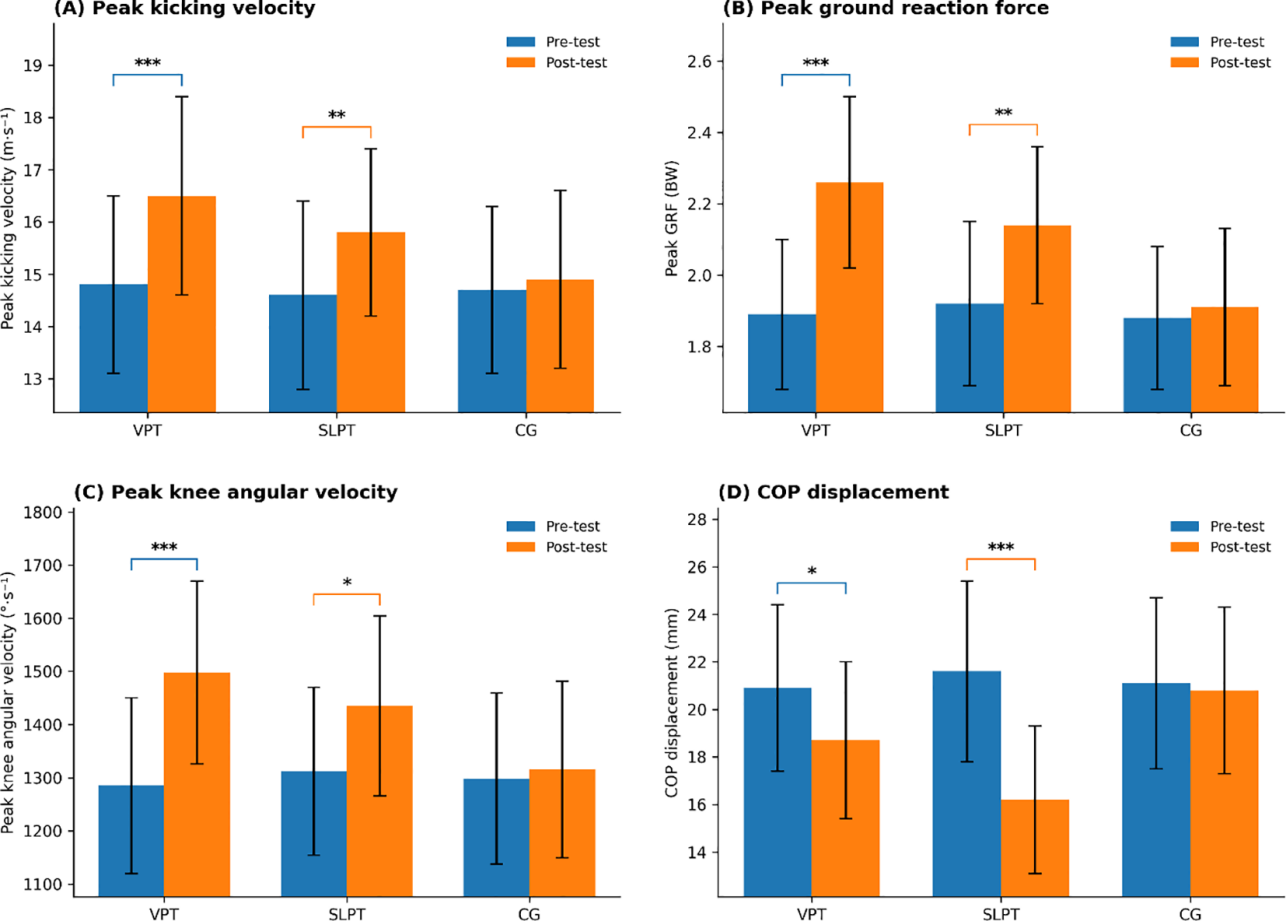
Comparative pre–post biomechanical adaptations in the VPT, SLPT, and CG groups. **(A)** Peak kicking velocity, **(B)** peak ground reaction force (GRF), **(C)** peak knee angular velocity, and **(D)** dynamic balance assessed by center of pressure (COP) displacement. *p < 0.05, **p < 0.01, and ***p < 0.001 indicate significant within-group differences from pre-test to post-test.

A significant Group × Time interaction was observed for peak GRF during kicking execution (F(2,33) = 9.42, p < 0.001, η^2^ = 0.36). Descriptive statistics for GRF and RFD are presented in **Table 3**, while the repeated-measures ANOVA results are summarized in **Table 4**. *Post-hoc* analysis revealed that both experimental groups demonstrated significant improvements compared with the control group. The VPT group showed the largest increase in peak GRF, improving from 1.89 ± 0.21 BW at pre-test to 2.26 ± 0.24 BW at post-test (p < 0.001, Cohen’s d = 1.05). The SLPT group also demonstrated significant improvement, increasing from 1.92 ± 0.23 BW to 2.14 ± 0.22 BW (p = 0.003, d = 0.79). In contrast, the control group showed no significant change (p = 0.41). Similarly, RFD exhibited a significant interaction effect (F(2,33) = 7.88, p = 0.001, η^2^ = 0.32). The VPT group demonstrated the largest increase in RFD (18.7%), followed by the SLPT group (12.3%), while the control group showed negligible change. Within-group effect sizes are presented in **Table 5**, whereas graphical comparisons of biomechanical changes are illustrated in **Figure 1B**. These results suggest that VPT produced greater improvements in bilateral explosive power, consistent with the training emphasis on vertical force production and stretch–shortening cycle efficiency.

**Table 3:** Descriptive statistics of biomechanical variables before and after the intervention.

Variable	Group	Pre-test (Mean ± SD)	Post-test (Mean ± SD)
Peak GRF (BW)	VPT	1.89 ± 0.21	2.26 ± 0.24***
	SLPT	1.92 ± 0.23	2.14 ± 0.22**
	CG	1.88 ± 0.20	1.91 ± 0.22
RFD (N·s^-1^)	VPT	3124 ± 356	3708 ± 402***
	SLPT	3189 ± 372	3582 ± 384**
	CG	3107 ± 341	3148 ± 359
Peak Kicking Velocity (m·s^-1^)	VPT	14.8 ± 1.7	16.5 ± 1.9***
	SLPT	14.6 ± 1.8	15.8 ± 1.6**
	CG	14.7 ± 1.6	14.9 ± 1.7
Peak Knee Angular Velocity (°·s^-1^)	VPT	1285 ± 165	1498 ± 172***
	SLPT	1312 ± 158	1435 ± 169*
	CG	1298 ± 161	1315 ± 166
Peak Hip Flexion Angular Velocity (°·s^-1^)	VPT	845 ± 110	964 ± 118**
	SLPT	862 ± 105	935 ± 114*
	CG	850 ± 112	861 ± 109
COP Displacement (mm)	VPT	20.9 ± 3.5	18.7 ± 3.3*
	SLPT	21.6 ± 3.8	16.2 ± 3.1***
	CG	21.1 ± 3.6	20.8 ± 3.5

Values are presented as mean ± SD. VPT, Vertical Plyometric Training; SLPT, Single-Leg Plyometric Training; CG, Control Group; GRF, ground reaction force; RFD, rate of force development; COP, center of pressure; BW, body weight. *p < 0.05, **p < 0.01, and ***p < 0.001 indicate significant within-group differences from pre-test to post-test.

**Table 4:** Two-way repeated measures ANOVA results for biomechanical variables.

Variable	Time effect F(p)	Group effect F(p)	Group × time interaction F(p)	Partial η^2^
Peak GRF	14.62 (<0.001)	5.88 (0.007)	9.42 (<0.001)	0.36
RFD	12.75 (<0.001)	4.91 (0.013)	7.88 (0.001)	0.32
Peak Kick Velocity	11.90 (<0.001)	4.55 (0.018)	8.61 (0.001)	0.34
Peak Knee Angular Velocity	10.44 (<0.001)	3.98 (0.028)	6.97 (0.003)	0.30
Peak Hip Angular Velocity	9.12 (0.004)	3.61 (0.037)	5.84 (0.006)	0.27
COP Displacement	15.31 (<0.001)	6.27 (0.005)	10.11 (<0.001)	0.38

GRF, ground reaction force; RFD, rate of force development; COP, center of pressure; η^2^, partial eta squared. Partial η^2^ interpretation thresholds were: small, 0.01, medium, 0.06, and large, 0.14. All interaction effects demonstrated large effect sizes, indicating substantial training-induced biomechanical adaptations.

**Table 5:** Within-group effect sizes (Cohen’s d) for training adaptations.

Variable	VPT	SLPT	CG
Peak GRF	1.05 (Large)	0.79 (Moderate–Large)	0.12 (Trivial)
RFD	0.97 (Large)	0.68 (Moderate)	0.09 (Trivial)
Peak Kick Velocity	0.98 (Large)	0.74 (Moderate)	0.11 (Trivial)
Peak Knee Angular Velocity	1.12 (Large)	0.73 (Moderate)	0.10 (Trivial)
Peak Hip Angular Velocity	0.84 (Large)	0.63 (Moderate)	0.08 (Trivial)
COP Displacement	0.52 (Moderate)	1.21 (Large)	0.07 (Trivial)

VPT, Vertical Plyometric Training; SLPT, Single-Leg Plyometric Training; CG, Control Group; GRF, ground reaction force; RFD, rate of force development; COP, center of pressure. Cohen’s d interpretation thresholds were: small, 0.2, moderate, 0.5, and large, 0.8. The largest effects were observed in explosive power variables in the VPT group and dynamic balance in the SLPT group.

### Changes in kick velocity

Kick velocity showed a significant Group × Time interaction (F(2,33) = 8.61, p = 0.001, η^2^ = 0.34). Pre- and post-test descriptive statistics for kick velocity are displayed in **Table 3**, while inferential statistics are summarized in **Table 4**. Both plyometric training groups demonstrated significant increases in kicking speed following the intervention. The VPT group improved kick velocity from 14.8 ± 1.7 m·s^-1^ to 16.5 ± 1.9 m·s^-1^ (p < 0.001, d = 0.98). The SLPT group improved from 14.6 ± 1.8 m·s^-1^ to 15.8 ± 1.6 m·s^-1^ (p = 0.002, d = 0.74). The control group showed no significant change (p = 0.47). Between-group comparisons revealed that post-intervention kick velocity in the VPT group was significantly higher than in the control group (p < 0.01), while the SLPT group also showed significantly greater velocity than the control group (p < 0.05). However, the difference between the VPT and SLPT groups did not reach statistical significance (p = 0.09). The magnitude of within-group adaptations is presented in **Table 5**, while visual changes in kick velocity are illustrated in **Figure 1A**. These findings indicate that both plyometric modalities enhanced the ability to generate high-speed kicking movements.

### Joint kinematics during kicking

Significant improvements were observed in joint angular velocity of the hip and knee joints following the intervention. A significant Group × Time interaction was identified for peak knee angular velocity (F(2,33) = 6.97, p = 0.003, η^2^ = 0.30). Descriptive statistics for knee and hip angular velocity are reported in **Table 3**, whereas ANOVA outcomes are presented in **Table 4**. The VPT group demonstrated the largest increase, from 1285 ± 165°·s^-1^ to 1498 ± 172°·s^-1^ (p < 0.001, d = 1.12). The SLPT group also showed improvement (1312 ± 158°·s^-1^ to 1435 ± 169°·s^-1^, p = 0.01, d = 0.73), whereas the control group showed no significant change. For hip flexion angular velocity, a significant interaction effect was also observed (F(2,33) = 5.84, p = 0.006, η^2^ = 0.27). Both experimental groups improved significantly, with the VPT group showing slightly greater increases. Effect size analysis is shown in **Table 5**, and graphical representations of knee angular velocity changes are displayed in **Figure 1C**. These results suggest that plyometric training enhanced the proximal-to-distal energy transfer along the lower-limb kinetic chain, contributing to faster kicking movements.

### Dynamic balance performance

Dynamic balance, assessed through COP displacement during single-leg support, demonstrated a significant Group × Time interaction (F(2,33) = 10.11, p < 0.001, η^2^ = 0.38). Descriptive statistics for COP displacement are presented in **Table 3**, while statistical interaction effects are summarized in **Table 4**. The SLPT group exhibited the greatest improvement in balance stability, with COP displacement decreasing from 21.6 ± 3.8 mm to 16.2 ± 3.1 mm (p < 0.001, d = 1.21). The VPT group also demonstrated a moderate improvement (20.9 ± 3.5 mm to 18.7 ± 3.3 mm, p = 0.04, d = 0.52). No significant change was observed in the control group (p = 0.56). Within-group effect sizes are provided in **Table 5**, whereas visual comparisons of balance adaptations are illustrated in **Figure 1D**. These findings indicate that SLPT was more effective in improving unilateral balance and postural control, which are essential for maintaining stability during kicking actions.

### Inter-limb asymmetry

The asymmetry index of unilateral force production significantly decreased in the SLPT group (from 12.4% to 7.1%, p = 0.002), indicating improved inter-limb symmetry. The VPT group showed a smaller reduction (11.9% to 9.8%, p = 0.08), while the control group remained unchanged. These findings further support the superior effectiveness of unilateral plyometric training for improving limb-specific neuromuscular control and movement symmetry.

## Discussion

The purpose of this study was to investigate the biomechanical adaptations of kicking performance following two different plyometric training modalities VPT and SLPT in competitive Wushu Sanda athletes. The findings demonstrated that both training interventions significantly improved key biomechanical variables associated with kicking performance, including peak GRF, RFD, peak kicking velocity, peak knee angular velocity, peak hip flexion angular velocity, and COP displacement. However, the results also revealed distinct adaptation patterns between the two training modalities. VPT produced greater improvements in explosive force production and peak kicking velocity, whereas SLPT resulted in superior improvements in dynamic balance and inter-limb stability. Meanwhile, the control group showed no meaningful changes across the measured biomechanical variables throughout the intervention period. These findings suggest that bilateral and unilateral plyometric training provide complementary neuromuscular adaptations that may collectively enhance sport-specific kicking performance in competitive Wushu Sanda athletes.

One of the primary findings of this study was the significant increase in peak GRF and RDF in both plyometric training groups, with the greatest improvements observed in the VPT group. These results are consistent with previous research indicating that VPT enhance the efficiency of the stretch–shortening cycle and improve neuromuscular force production capabilities (Bouteraa et al., 2020; Khlifa et al., 2015). The repetitive rapid eccentric–concentric muscle actions involved in vertical plyometric movements likely increased muscle–tendon stiffness and enhanced motor unit recruitment, allowing athletes to generate higher levels of force in a shorter period of time. From a biomechanical perspective, increased peak GRF during kicking suggest a more effective transfer of mechanical energy from the supporting leg to the kicking limb (Sasadai et al., 2015; Sørensen et al., 1996). This improved force generation capacity is critical in martial arts movements, where explosive lower-limb actions determine the velocity and impact force of strikes. The large effect sizes observed in explosive power variables in the VPT group further support the effectiveness of bilateral plyometric training for enhancing lower-limb power output in combat sport athletes.

Another important finding of the present study was the significant increase in kick velocity and joint angular velocity following the training intervention. Both experimental groups demonstrated improvements in peak hip and knee angular velocities, indicating enhanced coordination of the lower-limb kinetic chain during the kicking motion (Jung and Park, 2018). Efficient kicking performance relies on a proximal-to-distal sequence of segmental acceleration, in which the hip initiates the movement and mechanical energy is subsequently transferred through the knee and ankle joints. The observed increase in knee angular velocity suggests that plyometric training improved the athletes’ ability to rapidly accelerate the distal segments of the lower limb. This adaptation likely contributed to the significant increase in kick velocity observed in both training groups (Zhang et al., 2025). The greater improvements in kick velocity observed in the VPT group may be attributed to the bilateral force production characteristics of vertical plyometric exercises. These exercises emphasize maximal vertical impulse and rapid stretch–shortening cycle utilization, which may translate effectively to explosive sport-specific movements such as kicking.

In contrast to the explosive power adaptations observed in the VPT group, the SLPT group demonstrated the greatest improvements in dynamic balance performance, as reflected by the significant reduction in COP displacement during single-leg support phases. This finding highlights the importance of unilateral training stimuli for enhancing neuromuscular control and postural stability (Jung and Park, 2018). Kicking in Wushu Sanda is inherently a unilateral movement, requiring athletes to maintain stability on the supporting leg while performing high-speed movements with the kicking leg. SLPT impose greater demands on proprioceptive feedback, joint stabilization, and neuromuscular coordination (Gavagan and Sayers, 2017). These factors likely contributed to the substantial improvements in balance stability and inter-limb symmetry observed in the SLPT group. Improved dynamic balance may enhance movement efficiency and reduce unnecessary compensatory movements during kicking execution. Consequently, unilateral plyometric training may play an important role in optimizing the stability component of sport-specific motor performance in martial arts athletes.

An important practical implication of the present findings is that VPT and SLPT appear to produce complementary neuromuscular adaptations. While VPT primarily enhances explosive power and force production capacity, SLPT improves balance control and unilateral stability (Drouzas et al., 2020). Given that kicking performance requires both explosive force generation and dynamic balance, integrating both training modalities within a comprehensive strength and conditioning program may produce optimal performance outcomes. The distinct adaptation patterns observed in the VPT and SLPT groups suggest that bilateral and unilateral plyometric exercises may serve complementary roles in Wushu Sanda training. VPT appeared to primarily support explosive force production and kicking speed, whereas SLPT appeared to emphasize unilateral stability and dynamic balance. Therefore, coaches may consider these modalities according to the specific performance qualities they aim to develop.

The findings of this study provide several practical implications for coaches and strength and conditioning practitioners working with Wushu Sanda athletes. First, VPT such as squat jumps, countermovement jumps, and box jumps can be effectively used to improve explosive power and peak kicking velocity (Sørensen et al., 1996). Second, SLPT including single-leg hops, lateral bounds, and unilateral box jumps are particularly beneficial for enhancing dynamic balance and unilateral movement control. Therefore, incorporating both bilateral and unilateral plyometric exercises into Wushu Sanda training programs may lead to more comprehensive performance improvements. Coaches should also consider the specific demands of kicking techniques when designing plyometric training protocols, ensuring that exercises replicate the unilateral stability requirements inherent in martial arts movements.

Despite the important findings of this study, several limitations should be acknowledged. First, the sample size was relatively small and consisted primarily of university-level Wushu Sanda athletes, which may limit the generalizability of the findings to elite international competitors. Although gender distribution was reported and comparable across groups, the sample size was not sufficient to conduct adequately powered gender-specific subgroup analyses. Therefore, future studies with larger samples should examine whether sex-related differences influence biomechanical adaptations to plyometric training in Wushu Sanda athletes. Second, the study focused primarily on biomechanical variables related to kicking performance and did not directly measure impact force or scoring effectiveness in competitive matches. Future research should investigate the long-term effects of combined plyometric training programs that integrate both bilateral and unilateral exercises. Additionally, future studies could explore the relationship between biomechanical adaptations and actual competitive performance outcomes, such as strike effectiveness and scoring success in Wushu Sanda competitions.

## Conclusion

In conclusion, both VPT and SLPT significantly improved biomechanical variables associated with kicking performance in competitive Wushu Sanda athletes. VPT was more effective for enhancing explosive power and peak kicking velocity, whereas SLPT produced greater improvements in dynamic balance and unilateral stability. These findings highlight the importance of incorporating both bilateral and unilateral plyometric exercises into training programs to optimize sport-specific performance in martial arts athletes.

## Data Availability

The raw data supporting the conclusions of this article will be made available by the authors, without undue reservation.
